# Plastic maternal effects of social density on reproduction and fitness in the least killifish, *Heterandria formosa*

**DOI:** 10.1002/ece3.10074

**Published:** 2023-05-18

**Authors:** Samantha T. Levell, Samuel A. Bedgood, Joseph Travis

**Affiliations:** ^1^ Department of Biology Western Oregon University Monmouth Oregon USA; ^2^ Department of Integrative Biology Oregon State University Corvallis Oregon USA; ^3^ Department of Biological Science Florida State University Tallahassee Florida USA

**Keywords:** evolution, fish, *Heterandria formosa*, maternal effects, transgenerational plasticity

## Abstract

Environmental parental effects, also known as transgenerational plasticity, are widespread in plants and animals. Less well known is whether those effects contribute to maternal fitness in the same manner in different populations. We carried out a multigenerational laboratory experiment with females drawn from two populations of the least killifish, *Heterandria formosa,* to assess transgenerational plasticity in reproductive traits in response to differences in social density and its effects on maternal fitness. In the first and second generations, increased density decreased reproductive rate and increased offspring size in females from both populations. There were complicated patterns of transgenerational plasticity on maternal fitness that differed between females from different populations. Females from a population with historically low densities whose mothers experienced lower density had higher fitness than females whose mothers experienced higher density, regardless of their own density. The opposite pattern emerged in females from the population with historically high densities: Females whose mothers experienced higher density had higher fitness than females whose mothers experienced lower density. This transgenerational plasticity is not anticipatory but might be considered adaptive in both populations if providing those “silver spoons” enhances offspring fitness in all environments.

## Graphical abstract

Environmental parental effects, also known as transgenerational plasticity, are widespread in plants and animals. The extent to which those effects represent adaptive plasticity remains contentious. We executed a multigenerational laboratory experiment with females drawn from two populations of the least killifish, *Heterandria formosa*, to assess transgenerational plasticity in reproductive traits in response to a gradient in social density and assess whether any such plasticity might differ between two populations with historically distinct density regimes.

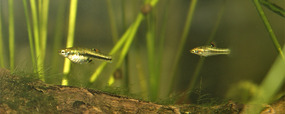

## 1 INTRODUCTION

Environmental parental effects, also known as transgenerational plasticity, are widespread in plants and animals (Bell & Hellmann, 2019; De Long et al., 2021; Donohue, 2009; Herman & Sultan, 2011; Meaney, 2001; Roach & Wulff, 1987; Rossiter, 1991; Weaver et al., 2004). Transgenerational plasticity has been demonstrated in response to a wide variety of ecological factors: temperature (Betini et al., 2020; Landy & Travis, 2018; Lee et al., 2020; Penney et al., 2021; Salinas & Munch, 2012; Sun et al., 2018; Tougeron et al., 2020), salinity (Griffiths et al., 2021), food availability (Kangassalo et al., 2020; Plaistow et al., 2006; Vega‐Trejo et al., 2018), exposure to predation risk (Hellmann et al., 2020; Lehto & Tinghitella, 2020; McGhee et al., 2021; Tariel et al., 2020), herbivory (Sobral, Sampedro, et al., 2021), and conspecific density (Langen et al., 2019; Li et al., 2019; Meise et al., 2016). However, the extent to which these effects represent adaptive plasticity between parent and offspring generations remains contentious (Marshall & Uller, 2007; Sanchez‐Tojar et al., 2020; Uller et al., 2013; Yin et al., 2019). There are compelling examples of adaptive plasticity (Allen et al., 2008; Galloway & Etterson, 2007; Kangassalo et al., 2020; Sun et al., 2018; Tougeron et al., 2020). However, many studies have either failed to find transgenerational plasticity where it was expected or found it to be maladaptive (Betini et al., 2020; Griffiths et al., 2021; Langen et al., 2019; Martin et al., 2019; Neylan et al., 2022; Penney et al., 2021). For any single ecological agent like conspecific density, some experiments demonstrate an adaptive effect (Langen et al., 2019) while others do not (Zipple et al., 2019).

One approach to understanding the effect of transgenerational plasticity on parental fitness is that of the reciprocal transplant, or a facsimile in experimental conditions (Hereford, 2009; Reznick & Travis, 1996). This approach assesses the relative fitness of individuals from two populations in each of two conditions, one condition representing an individual's own environment and the other representing a foreign environment. Reciprocal transplant approaches can be generic (exchanging individuals across different environments) or specific (exchanging individuals across a gradient of an individual environmental effect like temperature or population density). Such experiments can reveal how a trait affects fitness in contrasting environments and, in specific transplant experiments, whether selection on the trait changes based on environment.

Populations of the least killifish, *Heterandria formosa* (Pisces: Poeciliidae), in north Florida are ideally suited for this approach to studying transgenerational plasticity, particularly with respect to how maternal fitness (measured using replacement rate, which incorporates offspring size at birth to estimate offspring survival, per Henrich & Travis, 1988) might change with population density. Average population densities vary widely among locations, with remarkable long‐term consistency (MacRae & Travis, 2014). Density has a strong effect on maternal fitness (Leatherbury & Travis, 2019; Leips et al., 2009) and females from different populations have different norms of reaction of offspring number and size to variation in density (Leips et al., 2009). These differences are facilitated by the matrotrophic reproduction of *H. formosa*: Mothers provide almost all nutrition to the embryo through a placenta‐like ovary, which allows chemical communication between mother and embryo (Schrader & Travis, 2005, 2009, 2012b). Females also exhibit superfetation, which means they carry multiple broods of offspring at different developmental stages simultaneously. Because they reproduce continuously, there are continual opportunities for females to alter offspring size and number in response to environmental conditions, like changes in density or food level (Leips et al., 2009).

The least killifish is well‐suited for the multigenerational experiments necessary to quantify transgenerational plasticity and assess its significance (Burgess & Marshall, 2014). Individuals mature in 50–60 days (Felmy et al., 2021; Hale & Travis, 2015), females reproduce readily in suitable laboratory settings (Henrich & Travis, 1988), and transgenerational plasticity in juvenile growth rate in response to water chemistry and temperature has already been documented (Landy & Travis, 2018).

Here we report a comparative study of transgenerational effects in females from two populations, Wacissa River (WR) and Trout Pond (TP). These populations display characteristic differences in population density (Leips & Travis, 1999; MacRae & Travis, 2014; Richardson et al., 2006). Females from each population display genetically based life‐history differences that match predictions from density‐dependent selection (Leips et al., 2009; Schrader & Travis, 2012a). Individuals from WR are ~30% larger at maturity (Felmy et al., 2021) and have smaller broods of offspring that are, individually, ~50% larger in mass at parturition. Furthermore, females from TP display higher survival at low food densities than females from WR (Felmy et al., 2021). Our study is a facsimile of a reciprocal transplant: We examine transgenerational maternal effects in females living in densities like those they experience in nature (low in TP, high in WR) and like those unlike the densities they experience in nature (high in TP, low in WR). We predict female *H. formosa* will adjust offspring size and number in response to changes in social density. When social density is high, females should produce fewer, larger offspring. When social density is low, females should produce more, smaller offspring. Based on historical population densities in these populations, it is possible that we may see population‐specific responses; WR may adjust offspring size but not number in low social density conditions. Conversely, TP may have a maladaptive response to high density like in Leatherbury & Travis, 2019, and thus not be able to produce as many offspring in higher densities.

## 2 METHODS

### 2.1 Collection and husbandry

We collected *H. formosa* from WR and TP by means of dip netting in mid‐April 2015. While in the field, fish were treated with Furan for bacterial and fungal infections (1 ppt) and treated with Stress Coat and Amquel to reduce stress/ammonia production due to capture. We collected 112 female and 65 male *H. formosa* from each site (for a total of 354 fish) and held them in the collecting coolers for 1 week. The wild‐caught adult fish were actively reproducing when collected and continued in the coolers; we retrieved 225 TP and 85 WR juvenile fish from the coolers within 7 days of collecting the parents. We raised these F1 progeny at a density of 42–45 fish per 110‐liter aquarium (five aquaria for TP and two aquaria for WR). These aquaria had cover objects on the bottom and ample artificial vegetation for cover. We limited our recruitment of fish for the F1 generation to these fish in order to minimize any possible effects of parental age on the offspring they produced (Berkeley, 2004).

In our common gardens, the *H. formosa* began to mature at 42 days and were moved into adult density treatments at 75 days of age, which is when we started to see newborn juveniles in the common garden tanks. We fed experimental fish 10/mg/day/female of ground Tetramin® flake food per previous experiments (Leips et al., 2013; Schrader & Travis, 2009; Schrader & Travis, 2012a; Schrader & Travis, 2012b). By providing a constant per capita food level, we isolated, as much as possible, the effects of social density (the number of interacting individuals) from those of food limitation. Both factors contribute to the cumulative effects of density on maternal fitness (Leatherbury & Travis, 2019). While we cannot guarantee that all females consumed an equal amount of food or that any inequality in food consumption was the same at both densities, prior work has shown that this feeding regime does reveal the effects of social density independently of the effects of food limitation (Leatherbury & Travis, 2019). Prior work indicates that TP females experience greater mortality than WR females when per capita food is limited (Felmy et al., 2021). Because of this, we provided a constant, adequate level of per capita food at each density to preclude confounding population‐specific responses to food with population‐specific responses to social density.

We removed waste and uneaten food with nets three times weekly and changed 20% of the water each week. We added de‐ionized water as needed to replace water that had evaporated. We placed opaque plastic shields between aquaria so that individuals from different treatments were not able to see each other. We used artificial vegetation to provide approximately 50% cover of the water surface in each aquarium, a level consistent with natural cover (Richardson et al., 2006). We tested the water of a random subset of the aquaria weekly for nitrate, nitrite, and ammonia to ensure these values were close to zero throughout the duration of the experiment. We similarly sampled pH to ensure that there was a constant pH of around 7.8.

### 2.2 General experimental methods

We allowed neonates to grow to maturity in common garden, 110‐liter aquaria so that the experiment would manipulate only adult density. When newly matured fish were 75 days old, we assigned them at random to a treatment combination and transferred them to new 19‐liter aquaria. We assigned adult fish to “low” (L) or “high” (H) density treatments. High‐density treatments mimic typical densities in WR with a female‐biased sex ratio that is typical for these populations (Leips & Travis, 1999; Leips, Travis, & Rodd, 2000). We set the low density as low as was practical while still maintaining a female‐biased sex ratio. “High” density aquaria had five females and three males; “low” density aquaria had two females and one male. In terms of fish per liter, the low and high treatments translate into 0.16 and 0.42 fish per liter, respectively. For comparison with previous experiments using aquaria of the same size, our low density was comparable with the low densities in Leips et al. (2009) (0.19 fish per liter) and Leatherbury and Travis (2019) (0.16 fish per liter). Our high density was lower than the high densities of Leips et al. (2009) (0.57 fish per liter) and Leatherbury and Travis (2019) (0.58 fish per liter) so our difference between low and high densities was not as broad as the differences in these previous studies. For comparison with densities recorded in the natural populations (Leips & Travis, 1999; MacRae & Travis, 2014; Richardson et al., 2006), our low density is higher than the range of densities typically observed in TP (0.01–0.04 fish per liter) and higher than the lowest densities in WR (~0.08 fish per liter). Our high density is an order of magnitude higher than the highest densities typically observed in TP but comparable to the densities typically recorded in WR (~0.40 fish per liter). Our difference between low and high densities is thus much smaller than the differences between the natural populations in typical densities.

For assessing reproduction in the F1 generation, our design is a 2 × 2 factorial experiment, population (T for Trout Pond or W for Wacissa River) crossed with F1 adult density (L for low or H for high). For the F2 generation, we divided offspring from each combination of population and F1 density treatment into two further density treatments, low and high. This produced a 2 × 2 × 2 factorial design: population (T or W), F1 density (L or H), and F2 density (L or H). We denote individual treatment combinations by the three‐letter code in this order, so that T‐L‐H represents a replicate from Trout Pond with low F1 density and high F2 density.

Treatment conditions were maintained between generations but in two different locations. The first generation was conducted in the FSU Mission Road greenhouse. Fish from the second generation were moved to a vivarium on‐campus to maintain temperatures consistent with their summer breeding season in nature. We did this because the onset of autumn and winter conditions in the greenhouse would have caused a cessation of reproduction.

### 2.3 F1 methods

In this phase, we used 25 aquaria arranged in a stratified randomized block design throughout the greenhouse. (Population‐F1 Density): 8 at T‐L, 7 at T‐H, 5 at W‐L, and 5 at W‐H. We utilized all the fish we were able to; there were fewer replicates of WR because WR females have lower fecundity than TP females (Leips & Travis, 1999; Leips et al., 2009; Schrader & Travis, 2012a).

We allowed a 1‐week acclimation period for adults after we placed them in their assigned treatments. We then monitored reproduction for 48 days (six eight‐day periods or “weeks” to maximize reproductive potential), after which we euthanized the fish and saved them for other studies. A reproductive period of 48 days corresponds to the maximum female lifespan in WR (Travis, unpublished otolith data). We will refer to these 8‐day periods as weeks to simplify the discussion.

We removed all offspring from the F1 generation born during weeks one and two into separate 110‐liter aquaria to produce females for the F2 treatments and to keep rearing conditions consistent across generations. We kept offspring from a single aquarium together during the juvenile‐rearing phase. We set the F2 treatments using males and females from different parental aquaria in order to minimize inbreeding. We used offspring born in weeks one and two (roughly 16–24 days after initiation of the F1 treatments from the common garden‐rearing environment) for the F2 generation. It is worth noting that these offspring that did not experience full gestation in the adult treatments, which provides a conservative assay for any transgenerational effects.

We sacrificed fish born during week four to estimate the effects of our treatments on offspring size at birth, measured as dry mass. These fish had experienced their entire gestation period in the experimental treatments. We euthanized these neonates with MS‐222, preserved them in 5% formalin, and, eventually, freeze‐dried them and weighed them with a Kahn C‐31 microbalance.

### 2.4 F2 methods

Once the offspring from weeks 1 and 2 of F1 were mature and started giving birth at ~75 days of age, we moved them into treatment conditions for 6 weeks, just as we had done for the F1 generation. By this time, the experiment was in the on‐campus laboratory with constant summer temperature and light cycles. We kept these fish at 27.5°C on a 14–10 light cycle. Aquaria were set up identically to those in the greenhouse. In this phase, we had 19 aquaria arranged in a stratified randomized block design throughout the lab on two racks with three shelves per rack (Population‐F1 Density‐F2 Density): 3 at T‐L‐L, 2 at T‐L‐H, 2 at T‐H‐L, 4 at T‐H‐H, 2 at W‐L‐L, 2 at W‐L‐H, 2 at W‐H‐L, and 2 at W‐H‐H.

During this phase, we searched tanks twice weekly for juveniles. Because this was the last generation, all offspring produced in each of the 6 weeks were sacrificed on sight and freeze‐dried to determine the size (mass) at birth. The same processing methods used for the F1 offspring born in weeks three and four apply to all offspring produced from the F2 fish.

### 2.5 Statistical analysis

We examined weekly per capita offspring production rates in the F1 and F2 generations. In the F1 generation, we analyzed production rates in weeks 4–6. We used offspring born in the first 2 weeks to propagate the F2 generation. The offspring born in weeks 4–6 represent the first broods to have developed from fertilization to birth entirely in the experimental treatments, so they represented the full effect of those treatments. In the F2 generation, we analyzed offspring production rates in all weeks because we were not propagating a third generation. This also enabled us to assess whether transgenerational effects were expressed at the start of the F2 reproductive period, despite offspring is not having experienced full gestation in the experimental treatments, and if the strength of any such effects increased over time.

We analyzed weekly offspring production with a repeated measures analysis of variance with the offspring production from each aquarium as the response variable. Each aquarium was classified as a subject because individual offspring cannot be assigned to a specific mother. This means that we measure female fitness based on an average for all the females in a single replicate aquarium. The fixed treatment effects of population, density, and, for the F2 generation, F1 density were the between‐subject (between‐aquarium) effects and week, treated as a linearly ordered categorical variable, was the within‐subject (within‐aquarium) random effect. We began with a full model, containing all main effects and interactions, and used backward deletion of nonsignificant effects with *F*‐values below 1 to arrive at a final model. For the data in the F2 generation, we used an orthogonal polynomial contrast to test for a quadratic effect of week. We report results from the final model.

The analysis of offspring size at birth posed a more complicated challenge. First, whereas per capita production rates are characteristics of experimental populations, or aquaria, offspring size at birth is a measurement of an individual within a particular aquarium. This means that the mass of offspring born in the same aquarium in the same week is not independent of one another. This required including the identity of each aquarium as a random effect. Second, in the F2 generation, not all aquaria produced offspring in each week. This precluded a repeated measures approach. It also meant that an analysis of any particular week had to include at least two aquaria from each treatment combination to preclude conclusions being influenced by the overrepresentation of offspring from a single aquarium. Third, offspring mass generally decreased over time (measured in the weeks of the experiment), a pattern seen in other long‐term studies of reproduction in this species and attributable to sibling competition within a female (Schrader & Travis, 2012c).

We analyzed offspring size at birth separately in each week, specifically week 4 in the F1 generation and weeks 0–4 in the F2 generation. For analyses in the F2 generation, we pooled data from weeks 4 and 5 to ensure that each replicate contributed to this period. There were insufficient offspring from week 6 to include offspring born in that week from analyses of size at birth.

We used analysis of variance with the main effects of population, density, and, in the F2 generation, F1 density and all interactions. We nested aquarium identity within the two‐ or three‐way combinations of the fixed treatment effects. The effect of aquarium identity was always statistically significant and so we used the mean square for the effect of aquarium identity as the denominator for tests of the fixed effects and two‐way interaction. This is a conservative analysis. We began with a full model of all main effects and two‐way interactions and used backward elimination as described above for weekly per capita offspring production rates to arrive at a final model. We report the results of the final model.

Finally, for the F2 generation, we estimated maternal fitness by estimating the average replacement rate for females in each aquarium. Replacement rate uses both the size and number of offspring to determine the number of surviving offspring for a given female. This provides an estimate for the average F2 maternal fitness in order to examine transgenerational effects on maternal fitness. To do this, we combined data on weekly per capita offspring production with the average mass of offspring produced per week in each aquarium. Offspring survival to reproduction is an increasing function of offspring mass even in the laboratory (Henrich & Travis, 1988). Following Schrader and Travis (2009), we converted the average offspring mass into an average probability of survival. We used this number, together with the number of live offspring produced each week and the number of surviving females, to generate an estimate of net per capita production of surviving offspring or the replacement rate. We analyzed this variable with an analysis of variance with F2 density and F1 density as fixed treatment effects and using each individual aquarium as a replicate.

For each significant fixed effect in each analysis, we computed a standard measure of effect size, *ω* ^2^ (Olejnik & Algina, 2003). The quantity *ω* ^2^ varies between 0 and 1 for statistically significant effects and measures the strength of association between levels of an experimentally manipulated factor and the variation in a response variable, analogous to the proportion of variance explained in a regression analysis. In all cases, we checked the adequacy of our statistical models by plotting estimated values against actual values and residuals against estimates; we also checked assumptions by examining the distribution of residuals and checking for heterogeneity of variances. We removed 21 outlying data points from the final analyses of offspring mass because their studentized residual scores were above 3.0. These were most likely offspring that were overlooked in the week in which they were born and captured in a subsequent week after they had grown. We had 1376 offspring mass measurements (F1 and F2 combined); removing 21 removed about 1.5% of all measurements.

## 3 RESULTS

### 3.1 F1 generation

**FIGURE 1:**
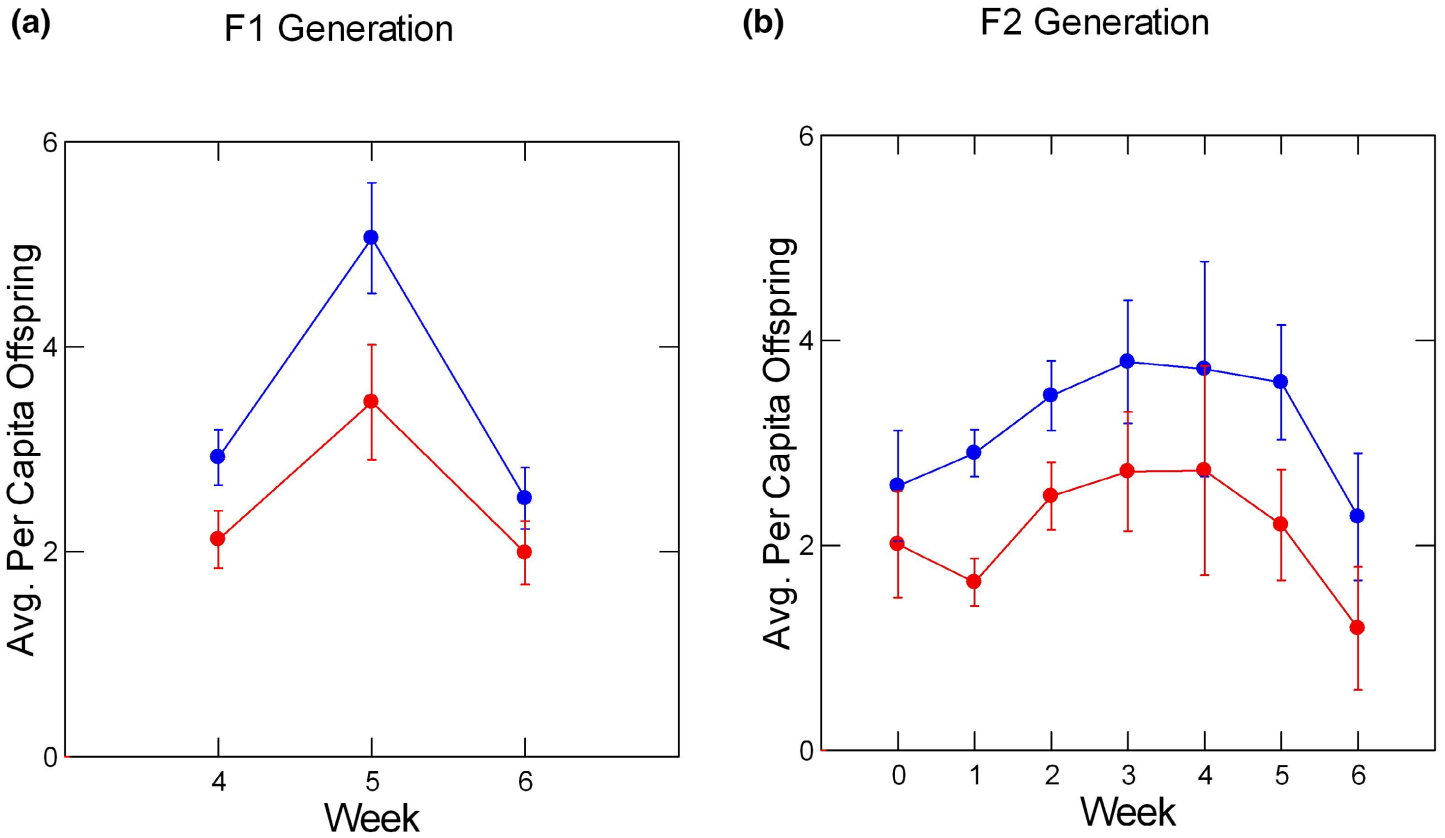
Least‐square means of per capita offspring production rates by week in the F1 generation (panel a) and the F2 generation (panel b), based on the final statistical model. Low‐density averages are depicted in blue, high‐density averages are depicted in red. Error bars signify ± one standard error.

**FIGURE 2:**
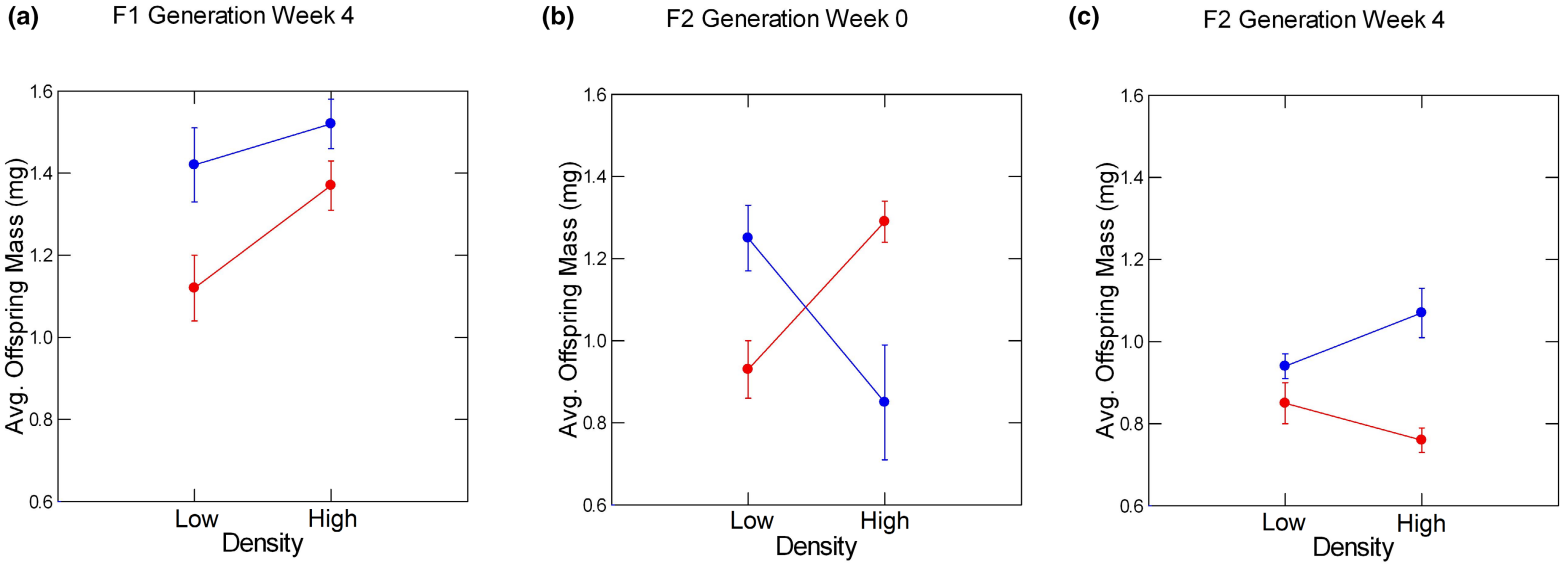
Least‐square means of offspring mass (in mg.) as a function of density and population identity, based on the final statistical model. Red circles indicate Trout Pond values, blue circles indicate Wacissa River values. (a) Values in week 4 for the F1 generation. (b) Values in week 0 for the F2 generation. (c) Values in week 4 for the F2 generation. All graphs are on the same scale; error bars signify ± one standard error.

The density manipulation was effective in causing significant changes in weekly per capita offspring production and offspring mass at birth in week 4 (Table 1; Figures 1a and 2a). While there were no differences in per capita offspring production between low‐ and high‐density aquaria in the first weeks, per capita offspring production increased markedly in weeks 4 and 5. In week 4, females at low density were producing about 3 offspring per female, on average, while females at high density were producing about 2 offspring per female. This difference increased in week 5, when females at low density were producing about 5 offspring per female, but females at high density were producing about 3.5 offspring per female. The difference declined in week 6. The repeated measures analysis of production in weeks 4–6 indicated a significant effect of week (Table 1). There was a significant effect of density but no difference between females from different populations and no interaction between population and density. There were no statistically significant interactions between the treatment effects and time, indicating that the effects of density and population identity did not change between weeks 4 and 6.

**TABLE 1:** Experimental factors retained in the final statistical model for each dependent variable in each generation.

Generation	Variable	Factor	*F*‐statistic	Degrees of freedom	*p*‐level	Effect size
F1	Offspring production	Week	13.49	2, 42	.001	NA
		Population	0.48	1, 21	.50	NA
		Density	12.15	1, 21	.002	0.16
		Population × Density	2.87	1, 21	.11	NA
	Offspring mass at week 4	Population	7.23	1, 14	.02	0.07
		Density	4.52	1, 14	.05	0.04
		Population × Density	1.33	1, 14	.20	NA
F2	Offspring production	Week	1.71	6, 72	.13	NA
		Quadratic trend in week	7.73	1, 72	.01	NA
		Population	6.09	1, 13	.028	0.17
		F2 Density	5.75	1, 13	.032	0.16
		F1 Density	0.70	1, 13	.42	NA
		Population × F2 Density	4.16	1. 13	.06	0.10
		Population × F1 Density	4.22	1, 13	.06	0.11
	Offspring mass at week 0	Population	0.11	1, 8	.75	NA
		F2 Density	0.01	1, 8	.92	NA
		Population × F2 Density	5.39	1, 8	.05	0.13
	Offspring mass at week 3	Population	6.94	1, 9	.025	0.09
	Offspring mass at week 4	Population	8.80	1, 11	.02	0.09
	Maternal fitness	Population	11.84	1, 13	.004	0.16
		F2 Density	25.80	1, 13	.001	0.37
		F1 Density	2.57	1, 13	.13	NA
		Population × F1 Density	13.71	1, 13	.003	0.17
		F2 Density × F1 Density	5.09	1, 13	.042	0.06

*Note*: Effect sizes are presented for significant (*p* ≤ .05) or nearly significant (*p* = .06) fixed effects. Interactions are denoted as “Factor 1 × Factor 2.” “NA” denotes not applicable.

The data on offspring mass at birth in week 4 represented 5 of the 8 TL aquaria, 5 of the 7 TH aquaria, and all 5 of the WL and WH aquaria. Offspring mass at birth at week 4 was 15%–25% larger in females from WR and about 7%–15% larger at the higher density (Figure 2a). Both differences were statistically significant but, considering the high variation in offspring mass within and among aquaria in the same treatment, both were weak effects. There was no interaction between population identity and density.

### 3.2 F2 generation

**FIGURE 3:**
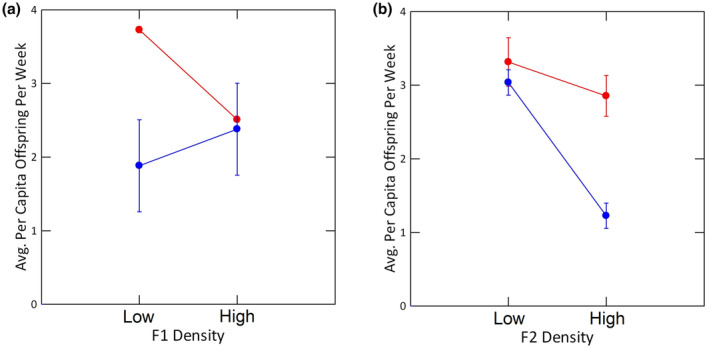
Least‐square means of weekly per capita offspring production in females from each population in the F1 generation (panel a) and the F2 generation (panel b). Values for Trout Pond, a low‐density population, are in red and values for Wacissa River, a high‐density population, are in blue. Error bars signify ± one standard error.

Offspring production rates rose from the start of reproduction through Week 2 and decreased steadily thereafter (Figure 1b). On a per capita basis, females at the higher density produced only 50% to 70% as many offspring weekly as females at the lower F2 density. The difference between the density treatments was smallest at the beginning of the experiment but increased immediately after the first week and persisted throughout the rest of the experiment. Females from WR produced fewer offspring each week; their per capita rate was 55%–70% of the rate exhibited by females from TP. The initial test for the overall effect of week was not significant (Table 1). However, the apparent rise and fall of the per capita reproduction rate over time was a significant one, as revealed by an a posteriori test for a quadratic trend in the data. Higher F2 density caused significantly lower production rates and females from TP produced significantly more offspring than females from WR. There was no indication of a main effect of F1 density. There was, however, a suggestion of interactions between population identity and F1 density (Table 1; Figure 3a) and population identity and F2 density (Figure 3b). In females from TP, higher F1 and F2 densities caused lower rates of offspring production, but the effect of density was stronger in the F1 generation. Females from WR displayed a different pattern; there was no significant effect of different densities in the F1 generation but a very strong negative effect of increased density in the F2 generation. None of the interactions of the fixed factors with week were significant, indicating that the differences between densities and populations were constant across the temporal trend of weekly changes.

At least two replicates from each of the 8 treatment combinations contributed data on offspring mass at birth in each of the weeks 0–4. We pooled data from weeks 4 and 5 to ensure that each replicate contributed to this period. There were insufficient offspring from week 6 to include offspring born in that week from analyses of size at birth. There were significant treatment effects on offspring mass at birth in three of the five periods (weeks: 0, 3, and 4). At the start of the experiment, there was a significant (Table 1) and reasonably strong interaction between population identity and F2 density (Figure 2b). Females from TP were producing larger offspring at the higher F2 density, as in the F1 results, but females from WR were producing smaller offspring at the higher density. This pattern gradually reversed such that by week 4, females from WR were making larger offspring at the higher F2 density while females from TP were making smaller offspring at the higher F2 density (Figure 2c). However, the high variation within and among individual aquaria precluded this interaction from being statistically significant. The only other statistically significant result for offspring mass was that females from WR made larger offspring than females from TP in week 3 and week 4/5.

**FIGURE 4:**
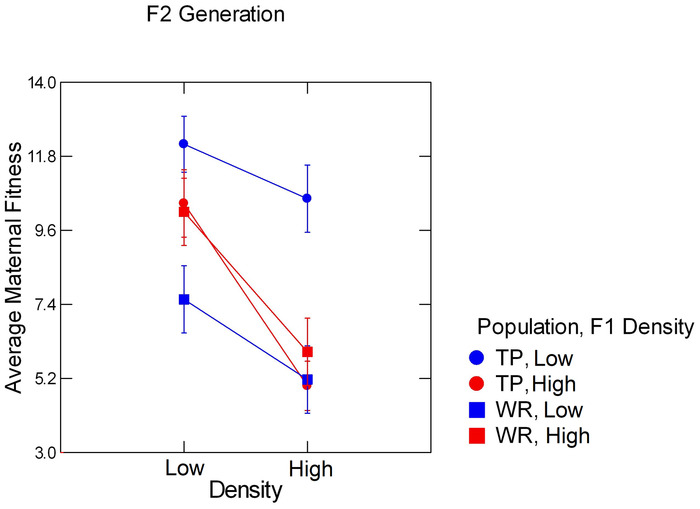
Least‐square means of maternal fitness in the F2 generation as a function of density in the F2 generation, population identity (circles denote Trout Pond, which is a low‐density population in nature; squares denote Wacissa River, which is a high‐density population in nature), and density of the mothers of the focal females (F1 generation: blue color indicates low density, red color indicates high density), based on the final statistical model. Error bars signify ± one standard error.

Combining offspring mass and offspring production rates into an estimate of maternal fitness revealed several striking results (Figure 4). There were two straightforward main effects. First, maternal fitness was about 60% lower at high F2 density than at low F2 density, regardless of population identity, a highly significant and strong effect (Table 1). Second, the average fitness of females from WR, by this measure was only about 75% that of females from Trout Pond, which was also a statistically significant effect, although not as strong as the effect of maternal density.

There was clear evidence of a strong, complicated transgenerational effect of F1 density that was mediated through interactions with population identity and F2 density (Figure 4). First, there was a statistically significant interaction of population identity and F1 density (Table 1). Trout Pond females whose mothers experienced low F1 density had a 20%–25% higher maternal fitness, on average, than TP females whose mothers experienced high F1 density, regardless of their own F2 density. Conversely, Wacissa River females whose mothers experienced low F1 density had ~15% lower maternal fitness, on average, than females whose mothers experienced high F1 density. This was most evident at the low F2 density, but the direction of difference is the same at the high F2 density. There was a statistically significant but weak interaction between F1 and F2 densities (Table 1): Females from both populations whose mothers experienced high F1 densities were more sensitive to the depressant effects of higher F2 density than females whose mothers experienced low F1 densities. In all, the manipulated factors explained approximately 75% of the total variance in maternal fitness in this phase of the experiment.

## 4 DISCUSSION

The results of our experiment reiterate a trade‐off between offspring size and number occurs at two levels in these populations. Schrader and Travis (2012c) showed that, in populations where females made larger offspring, they made fewer of them per brood. Leips et al. (2009) showed that females from both of these populations displayed phenotypic plasticity of offspring size and number to density. However, the nature of the trade‐off had evolved differently in each population. Females from WR responded to higher density with larger declines in offspring size and smaller declines in offspring number, whereas females from TP responded more in offspring number than offspring size.

Two of the results reported here are novel. First, in the F2 generation, the change in offspring size at birth with time differed between WR and TP females. Offspring produced by WR females were, initially, equal in size at both densities. By weeks 4/5, WR females at higher density were producing larger offspring than females at lower density, as observed in previous work. By contrast, TP females were producing larger offspring at higher rather than lower density at the start. By week 4/5 they were producing smaller offspring at the higher density. While prior work by Leatherbury and Travis (2019) did not take time explicitly into account, they also found that wild‐caught TP females produced smaller offspring at higher densities. Second, we found evidence of a complicated transgenerational effect of F1 density on maternal fitness. The strength of this effect was decidedly greater in TP females than WR females. This result is similar to results on transgenerational plasticity in growth rate and male body shape (Landy & Travis, 2018): Growth and shape of TP males displayed stronger environmental maternal effects across generations than did WR males. In our experiment, there was no indication that the effect of the F1 density on offspring production rate decreased over time within a generation, so it is fair to regard this effect as a lasting one and not transient. In nature, female *H. formosa* lifespan is around 7–9 weeks (Travis, unpubl. Data). Furthermore, population density tends to vary on a month‐to‐month basis in low‐density populations (like TP) but less so in high‐density populations (like WR) (Richardson et al., 2006).

Several results we see regarding population differences in size and number of offspring were expected based on prior results. The higher offspring production rates of TP females after two generations in a common environment with WR females is the same effect observed in earlier genetic studies (Leips, Travis, & Rodd, 2000). The decrease in individual offspring mass at birth over time reflected the rate of decrease seen in experiments in which individual females were the units of observation (Cheong et al., 1984; Travis et al., 1987) and has been ascribed to the effects of sibling competition (Schrader & Travis, 2012c). The generally larger offspring produced by WR females, in comparison with TP females, reflects population differences observed in natural populations (Leips & Travis, 1999; Schrader & Travis, 2012a) and mesocosm experiments (Leips et al., 2009). The increased mass of offspring at birth from WR females at the higher density is the same effect observed previously (Leips et al., 2009).

Our design admittedly confounds social density with the opportunity for sexual selection and polyandry (note that sex ratios are similar: 0.33 in the lower density and 0.37 in the higher density). As detailed in our methods, we set the high density to mimic typical densities in WR with a female‐biased sex ratio that is typical for these populations (Leips & Travis, 1999; Leips, Travis, & Rodd, 2000). Most theory for the effects of sexual selection and polyandry on reproductive rates posits that each process should increase those rates (Arbuthnott et al., 2015; Thonhauser et al., 2019). In nature, higher densities are associated with lower reproductive rates (Leips & Travis, 1999; Schrader & Travis, 2012a) and, in previous experimental studies, higher density depressed reproductive rates (Leatherbury & Travis, 2019; Leips, McManus, & Travis, 2000). Because the higher density allows for mate choice between males and/or polyandry, a positive effect from these forces will act counter to the direct effects of a higher density.

The direct effects of density on offspring production in both generations and the effect of parental density on maternal fitness likely emerged from the stressful effects of social interactions under crowded conditions (Leatherbury & Travis, 2019). Aggression between females was observed in the experimental tanks, but we did not quantify it. Increased cortisol levels have been found in the ovaries of damselfish that experienced high social densities. Furthermore, offspring size was inversely correlated with maternal density, suggesting crowded, stressed mothers produce smaller offspring (McCormick, 2006). While our results demonstrate that social density plays a prominent role in driving these transgenerational effects, they do not preclude a role for food limitation per se. Although we provided a constant amount of per capita food at both densities, we could not guarantee that all females in an aquarium consumed an equal ration of food or that any inequality was not increased at the higher density. However, prior work (Leatherbury & Travis, 2019) demonstrated strong effects of social density that were independent of the level of per capita food provided. In that light, our results open a new avenue of research into how stressful social interactions might translate into transgenerational effects (Sobral, Neylan, et al., 2021; Webster et al., 2018).

It is difficult to ascertain from these data alone whether the transgenerational plasticity in maternal fitness in these data is an adaptive response to the social density regime. We studied only one low‐density and one high‐density population, so we cannot ascribe the different responses in each population to divergent adaptations to social density. We can examine the consequences of maternal density in each population without drawing conclusions about the driving force of their idiosyncratic responses. The pattern in TP resembles a “silver spoon effect” (Grafen, 1988); TP females whose mothers experienced low density displayed higher fitness than TP females whose mothers experienced high density regardless of their own social density experience. We can speculate that the high‐density conditions may have been stressful to TP fish, especially considering the effects of food availability were controlled, but we cannot say conclusively. The situation is less clear about the effects on females from WR. In this case, females whose mothers experienced high density had higher fitness than females whose mothers experienced low density, a silver spoon from the opposite direction. The effect was pronounced at low density; whether one accepts it at high density depends on whether one looks at the pattern or the statistical significance of the difference between the two types of females at high density. High density is typical of WR, but we cannot isolate this as the reason for the fitness trade‐off without incorporating more high‐ and low‐density populations. Another factor that influences natural differences in the natural density of *H. formosa* population is predation, which may contribute to responses to social density.

Even with this liberal interpretation of the silver spoons, there is a further argument against an adaptive interpretation. Females from both populations whose mothers experienced high density were much more sensitive to the depressant effects of high F2 density than females whose mothers experienced low density (the F1 × F2 interaction in maternal fitness). An adaptive explanation would predict exactly the opposite pattern, that maternal experience at high density should buffer offspring sensitivity to high density.

Our work contributes to expanding literature documenting that transgenerational plasticity can differ substantially among populations (Matesanz et al., 2022; Sultan et al., 2009) and even among genotypes (Alvarez et al., 2020; Latzel et al., 2022). Whether such differences reflect local adaptation in maternal responses to the environment remains to be determined. Only about half of reciprocal transplant experiments for conventional traits produced compelling evidence of local adaptation (Hereford, 2009). Making the case for a complex trait like a transgenerational effect is more demanding. However, documenting idiosyncratic responses in a reciprocal transplant facsimile is a necessary first step. Integrating those responses with the long‐term regimes of environmental variation and predictability (Burgess & Marshall, 2014; Ezard et al., 2014; Joschinski & Bonte, 2020; Leimar & McNamara, 2015) is the next, more difficult step.

## AUTHOR CONTRIBUTIONS

**Samantha T. Levell:** Conceptualization (equal); data curation (equal); formal analysis (equal); funding acquisition (equal); investigation (lead); methodology (equal); project administration (lead); resources (equal); supervision (equal); validation (equal); visualization (equal); writing – original draft (equal); writing – review and editing (equal). **Samuel A. Bedgood:** Formal analysis (equal); investigation (supporting); project administration (supporting); resources (supporting); supervision (supporting); writing – review and editing (supporting). **Joseph Travis:** Conceptualization (equal); data curation (equal); formal analysis (lead); funding acquisition (equal); investigation (equal); methodology (equal); project administration (equal); resources (equal); software (equal); supervision (equal); validation (equal); visualization (equal); writing – original draft (equal); writing – review and editing (equal).

## CONFLICT OF INTEREST STATEMENT

None of the authors declare any conflicts of interest regarding this manuscript or the work it contains.

## Data Availability

The data that support the findings of this study are openly available in DRYAD at http://doi.org/10.5061/dryad.n8pk0p319.
